# Transactions of the College of Physicians of Philadelphia

**Published:** 1876-01

**Authors:** 


Transactions of the College of Physicians of Philadelphia.

This little volume of two hundred pages contains a number of valuable papers. Among others, we have read with pleasure Dr. S. Weir Mitchell's paper on the use of the nitrite of amyl in the various forms of spasms, and the use of the same remedy by Dr. Forbes in acute tetanus.

A prominent feature of the volume is the report of the autopsy of the bodies of Chang and Eng Bunker, commonly known as the Siamese Twins, by Drs. Pancoast and Allen. The volume clearly indicates the college a working body.

